# Management of the Brain: Essential Oils as Promising Neuroinflammation Modulator in Neurodegenerative Diseases

**DOI:** 10.3390/antiox13020178

**Published:** 2024-01-31

**Authors:** Rosanna Avola, Alessandro Giuseppe Furnari, Adriana Carol Eleonora Graziano, Alessandra Russo, Venera Cardile

**Affiliations:** 1 Faculty of Medicine and Surgery, University of Enna “Kore”, 94100 Enna, Italy; rosanna.avola@unikore.it; 2 Department of Biomedical and Biotechnological Sciences, University of Catania, 95123 Catania, Italy; alessandro.furnari@studium.unict.it; 3 Department of Drug and Health Sciences, University of Catania, 95123 Catania, Italy; alrusso@unict.it

**Keywords:** neuroinflammation, oxidative stress, essential oils, natural compounds, neurodegenerative diseases, antioxidants

## Abstract

Neuroinflammation, a pivotal factor in the pathogenesis of various brain disorders, including neurodegenerative diseases, has become a focal point for therapeutic exploration. This review highlights neuroinflammatory mechanisms that hallmark neurodegenerative diseases and the potential benefits of essential oils in counteracting neuroinflammation and oxidative stress, thereby offering a novel strategy for managing and mitigating the impact of various brain disorders. Essential oils, derived from aromatic plants, have emerged as versatile compounds with a myriad of health benefits. Essential oils exhibit robust antioxidant activity, serving as scavengers of free radicals and contributing to cellular defense against oxidative stress. Furthermore, essential oils showcase anti-inflammatory properties, modulating immune responses and mitigating inflammatory processes implicated in various chronic diseases. The intricate mechanisms by which essential oils and phytomolecules exert their anti-inflammatory and antioxidant effects were explored, shedding light on their multifaceted properties. Notably, we discussed their ability to modulate diverse pathways crucial in maintaining oxidative homeostasis and suppressing inflammatory responses, and their capacity to rescue cognitive deficits observed in preclinical models of neurotoxicity and neurodegenerative diseases.

## 1. Introduction

Neuroinflammation is a complex innate immune response occurring in the central nervous system (CNS), orchestrated primarily by specialized resident cells, notably glial cells, with microglia and astrocytes taking center stage. Pathogen-associated molecular patterns (PAMPs) and damage-associated molecular patterns (DAMPs) trigger neuroinflammation as they are recognized by pattern recognition receptors (PRRs) expressed in microglia, which, as a consequence, activate their phagocytic capabilities and the release of signaling molecules that mediate the neuroinflammatory response by allowing the activation and recruitment of other immune cells in situ. This physiological immune process aims to protect against pathogens or damaged cells. However, when uncontrolled and prolonged, it can lead to neuronal death and neurodegeneration.

To degrade phagocytosed material, activated microglia generate neurotoxic reactive oxygen species (ROS) and reactive nitrogen species (RNS), resulting in harmful effects on neural tissue. In addition, pro-inflammatory molecules might stimulate neuronal cell death and increase blood–brain barrier permeability, disrupting its integrity [1,2,3]. Neuroinflammation is a common feature of many brain diseases such as Alzheimer’s disease, Parkinson’s disease (PD), amyotrophic lateral sclerosis (ALS), frontotemporal lobar dementia (FTLD), Huntington’s disease (HD) and multiple sclerosis (MS); therefore, targeting its mechanisms might unveil new promising therapeutical strategies in their management [2,4,5].

While pharmacological treatments are available, there is an ongoing requirement for the development and discovery of new effective biomolecules that can enhance the quality of life for individuals afflicted by these diseases. Regarding this matter, a substantial body of research has been published on the advantageous neuroprotective effects of natural compounds that target this innate immune process [6,7]. Among all, phytocompounds within essential oils have demonstrated a promising capacity to counteract neuroinflammation and oxidative stress in preclinical models of neurotoxicity and neurodegenerative diseases, along with a significant improvement in cognitive processes such as learning and memory in these experimental systems [8,9].

**Figure 1:**
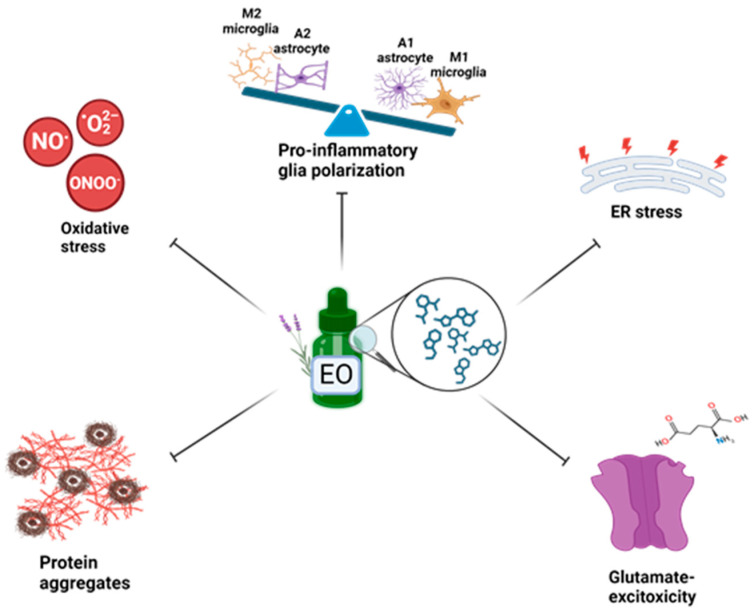
Scheme of the potential mechanisms of essential oils in promoting neuroprotection through the inhibition of processes implicated in neuroinflammation (created with BioRender.com, accessed on 10 January 2024).

This review places its focus on the role of neuroinflammation in the development and progression of neurodegenerative diseases and the potential benefit of essential oils (or their components) in their treatment and prevention (Figure 1).

## 2. Main Mediators of Neuroinflammation: The Role of Microglia and Astrocytes

**Figure 2:**
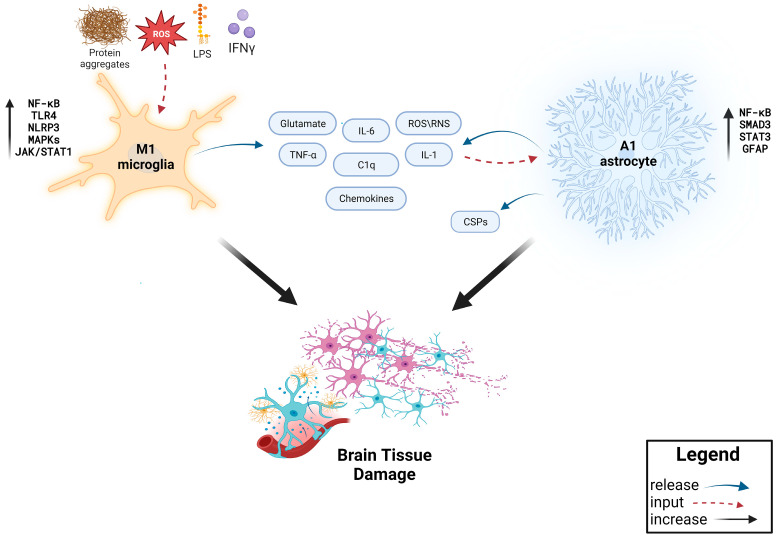
Scheme of molecular events that regulate inflammatory response in the brain (created with BioRender.com, accessed on 10 January 2024).

Microglia and astrocytes are two key cellular regulators of inflammatory processes developing in the CNS. These two cell types can either exert pro-inflammatory or anti-inflammatory functions according to their polarization, classically categorized as M1 (pro-inflammatory) or M2 (anti- inflammatory) for microglia and A1 (pro-inflammatory) or A2 (anti-inflammatory) for astrocytes. Remarkably, many neuroprotective natural compounds function by rebalancing the pro-inflammatory phenotypes toward the anti-inflammatory ones [10,11]. It is important to underscore that categorizing these cells in this binary manner might not accurately represent the diverse phenotypes of microglia and astrocytes; thus, it is noteworthy to view them as existing along a spectrum rather than as entirely separate populations [2,12,13]. However, in this review, we employed the traditional dichotomous classification of microglia and astrocytes to examine their respective roles in neuroinflammation (Figure 2).

### 2.1. Microglia

Microglia, the macrophage-lineage cells of the CNS, represent the initial cell type that reacts to danger. These immune cells can shift from one phenotype to another in response to distinct environmental conditions within the CNS. When exposed to PAMPs or DAMPs, such as lipopolysaccharide (LPS) or reactive species, respectively, as well as IFN-γ, microglial cells are activated in the M1 phenotype, expressing pro-inflammatory signatures such as interleukin (IL)-1β, IL-6, tumor necrosis factor (TNF)-α, chemokines, inducible nitric oxide synthase (iNOS), adenine dinucleotide phosphate (NADPH) oxidase and cyclooxygenase (COX)-2 [12,14]. This pro-inflammatory phenotype is associated with neural tissue damage [15]. NADPH oxidase produces ROS, such as superoxide anions, which in turn can combine with nitric oxide (NO) generated by iNOS to form peroxynitrite radicals [16]. When their concentration is greater than the cellular antioxidant capacity, reactive species are toxic, as they are capable of inducing DNA and protein damage as well as oxidizing the cellular membrane, resulting in lipid peroxidation and disruption of its properties, leading to necrotic cellular death [15,16,17]. TNF-α stimulates microglia in an autocrine manner, leading to an excessive release of glutamate, which consequently causes excitotoxic neuronal cell death [18]. Excessive glutamate dysregulates Ca^2+^ influx in neurons through hyper stimulation of the NMDA receptor, which in turn leads to RNS and ROS production, determining cellular death and exacerbating the neuroinflammatory response [19]. Several pathways are related to the pro-inflammatory switch of microglia.

LPS is recognized by, and thus activates, the Toll-like receptors (TLR)-4 signal pathway, which in turn culminates in a pro-inflammatory cascade and M1 microglia polarization. TLR4, activated by its ligand, leads to an enhanced activity of nuclear factor kappa-B (NF-κB) and mitogen-activated protein kinases (MAPKs) JNK, ERK and p38, which enhance pro-inflammatory mediator transcription [7,12,20]. ROS and ion fluxes can trigger NOD-like receptor pyrin-domain-containing 3 (NLRP3)-inflammasome signaling and, hence, the neurotoxic M1 activation of microglia [21,22]. Of note, the NLRP3 inflammasome pathway is associated with the induction of a pro-inflammatory form of programmed cellular death called pyroptosis [23]. The Janus kinase/signal transducer and activator of transcription 1 (JAK1-2/STAT1) pathway is likewise implicated in M1 polarization. IFN-γ acts through this pathway, and when it binds to its receptor, it triggers STAT1 phosphorylation, increasing its transcriptional activity, which in turn upregulates pro- inflammatory genes [12,24,25]. Ultimately, contact-dependent Notch signaling drives microglia to the pro-inflammatory phenotype [26,27].

Given the pro-inflammatory role of the aforementioned signaling pathways, their pharmacological targeting may be beneficial in regulating the shift of microglia from the M1 to M2 polarization state. This modulation holds promise for the treatment of a range of brain diseases [28,29].

As already mentioned, induced by cytokines such as IL-4, IL-13 and IL-10, microglia can exhibit an anti-inflammatory phenotype. M2-polarized microglial cells release anti-inflammatory cytokines such as IL-10, Transforming Growth Factor (TGF)-β and IL-1R antagonists, which collectively act in opposition to their pro-inflammatory counterparts [30,31]. Additional factors expressed by M2 microglia associated with the resolution of neuroinflammation and neuroprotection include Arginase1 (Arg1), which suppresses NO production by competing with iNOS for arginine as a substrate and determines the production of molecules (i.e., polyamines) involved in tissue repair, cell proliferation and survival [30,32,33]; CD206 (also known as macrophage mannose receptor 1), a phagocytic receptor that binds myeloperoxidase and lysosomal hydrolases and, because of that, plays a pivotal role in the resolution of neuroinflammation [30,34]; and neurotrophic factors such as BDNF [30]. Several pathways are positively associated with M2 polarization. Some examples comprise JAK1/STAT6 (triggered by IL-4), the cannabinoid receptor 2 (CB2)/peroxisome proliferator-activated receptor gamma (PPAR-γ) axis [12,35,36], triggering receptor expressed on myeloid cells 2 (TREM2) signaling [20,37,38] and the PI3K/Akt cascade [39]. Notably, the role of this latter pathway on M1 to M2 polarization seems to be a function of specific Akt isoforms [40]. Boosting these pathways or, conversely, inhibiting the pro-inflammatory ones with exogenous compounds could be beneficial in the treatment of neuroinflammation associated with various brain disorders.

### 2.2. Astrocytes

Astrocytes play a fundamental role in maintaining brain homeostasis and are thus implicated in many CNS disorders. Notably, they contribute to blood–brain barrier (BBB) integrity, neuronal metabolism, synapse and neurotransmission regulation, potassium clearance, glymphatic flow control and host-defense mechanisms [41,42,43]. In the context of neuroinflammation, microglia and astrocytes interact to modulate the course of the response to an insult. Pro-inflammatory signals released by M1 microglia such as TNF-α, IL-1α and C1q stimulate reactive A1 astrocytes [44]. This particular astrocytal polarization exhibits a shared profile of secreted molecules to that of pro-inflammatory microglia, thus contributing to the augmentation of the neuroinflammatory burden [45,46].

Pointing out the remarkable role of microglia interaction with astrocytes in their A1 polarization, knock-out mice lacking microglia failed to induce A1 astrocytes after LPS treatment, while wild-type showed strong A1 induction [44]. Furthermore, it was demonstrated that the NLRP3 inflammasome pathway in microglia rather than in astrocytes is strongly associated with their pro-inflammatory shift and, accordingly, the knock-out of NLRP3 in microglia mitigates the neuronal dysfunction provoked by A1-like astrocytes, both in in vitro and in vivo settings [47,48]. A1 astrocytes release neurotoxic factors and promote glial scar formation, which overall can exert detrimental outcomes for brain repair and neuronal cell survival. For example, glial fibrillary acidic protein (GFAP) and chondroitin sulfate proteoglycans (CSPGs), the main components of glial scars, inhibit axonal regeneration [13,44,49,50]. Different signaling pathways and transcription factors are involved in glial scar formation driven by reactive astrocytes, including IL- 1/NF-κB, IL-6/STAT3, NOTCH/STAT3 and TGF-β/SMAD3; therefore, attenuating this neuroinflammatory-related process targeting these cell signaling cascades might be of benefit in CNS pathologies [51,52,53]. Analogously, M2 microglial cytokines trigger reactive A2 astrocytes, associated with anti-inflammatory cytokine release, neuroprotection and repair [44,45,46,54]. Pathways and transcription factors that are linked with enhanced A1 to A2 polarization include the PI3K/Akt axis [55] and STAT6, with this latter being associated with the upregulation of antioxidants genes in A2 astrocytes, such as nuclear factor erythroid 2-related factor 2 (Nrf2) and Arg1 [56].

It is worth emphasizing that comprehending the mechanisms implicated in the regulation of neuroinflammation holds significant relevance in order to modulate this immune response through external molecules.

## 3. Neuroinflammation in Neurodegenerative Diseases: An Immunological Perspective

Neurodegeneration is a characteristic of numerous brain pathologies in which CNS functions deteriorate over time [5].

Neurodegenerative diseases place a significant burden on developed societies with aging populations. While research in this field is highly active, there remains a deficiency in comprehending the etiopathogenesis of these disorders, which is crucial for the discovery of new molecular targets and the development of therapies. In recent years, the role of neuroinflammation has emerged as a distinguishing feature of neurogenerative diseases, making it a new promising molecular process to focus on [5,57,58].

Notably during aging, a major risk factor for the development of neurodegenerative diseases, inflammatory processes rise in the brain (inflammaging) and there is a tendency for an increase in microglia M1/M2 ratio [33,59]. Additionally, the upregulation of gene signatures of M1-polarized microglia has been found in post-mortem tissues of patients with neurodegenerative disorders [60].

Along the same lines, an increase in A1 pro-inflammatory astrocytes has been identified in post-mortem neural tissues of individuals affected by AD, HT, PD, MS and ALS [44]. These findings suggest that neurodegenerative diseases develop in the context of a neuroinflammatory microenvironment.

Protein aggregates within neurons and in the extracellular space are a common characteristic and a hallmark of neurodegenerative diseases such as PD, AD, FTLD, HD and ALS [5,61]. Various studies clearly showed a link between protein aggregates in neurodegenerative diseases and neuroinflammation. Here, some examples of recent research and findings about this intricate association are provided.

Alpha-Synuclein (αSyn) aggregates (a pathological hallmark of PD) have been shown to lead to neuroinflammation and neurodegeneration through double-stranded DNA breaks and the induction of the DNA sensor GMP-AMP synthase (cGAS)/stimulator of interferon genes (STING) immune pathway, in a microglial and astrocyte mixed culture and a mouse model of PD [62]. Furthermore, in this study, the authors found STING upregulation in autopsied PD patients relative to healthy controls. These findings (associated with the amelioration of motor dysfunction symptoms in mice with STING knock-out) imply that this immune pathway, and therefore neuroinflammation, is an important feature in a neurodegenerative disorder such as PD [62,63]. Similarly, in an αSyn-driven mouse model of PD, the NLRP3 inflammasome cascade has been found to be upregulated in microglia [64]. The NLRP3 inflammasome pathway is also upregulated in microglia of mice models of PD driven by mitochondrial dysfunction and oxidative stress (thus independently of αSyn aggregates) and it precedes dopaminergic neuron degeneration and motor deficits [64]. Accordingly, NLRP3 pharmacological inhibition was found to be neuroprotective in these PD mice models. Furthermore, in PD patients, the upregulation of inflammasome markers has been evidenced in the substantia nigra of post-mortem brains [64]. Thus, taken together with other similar studies, these observations indicate that the aforementioned inflammatory signaling pathway is a key feature of this neurodegenerative disease [64,65]. Beta-amyloid (Aβ) aggregates, an AD pathological hallmark, induce the NLRP3 pathway through TLR4 in the BV-2 microglia cell line, and its conditioned medium reduces HT-22 neuronal cell line viability [66]. In another research study investigating the activation mechanism of the NLRP3 pathway by Aβ in primary microglia, the results revealed that Aβ aggregates initiate a process that involves the spleen tyrosine kinase (Syk)-mediated inactivation of AMPK. This inactivation leads to mitochondrial stress and an increase in the production of ROS, which subsequently triggers the NLRP3 cascade [67]. Interestingly, and in alignment with these findings, markers of pyroptosis (i.e., cleaved gasdermin D), an NLRP3-related inflammatory type of programmed cellular death, has been found to be upregulated in the post-mortem AD brain [68]. Moreover, the upregulation of genes and proteins associated with both inflammasome activation and pyroptosis is also observed in CNS post-mortem tissues of individuals with ALS and MS [69,70], underscoring the pivotal role of these processes as hallmarks in the landscape of neurodegenerative diseases. Of note, acetylcholinesterase (AChE) favors Aβ aggregate formation, thereby decreasing its activity associated with the inhibition of Aβ-fibrillogenesis [71].

Taking into account the central role of neuroinflammation in the abovementioned neurodegenerative diseases (described as examples of the various other brain disorders with neuroinflammation as a characteristic component), targeting immune pathway effectors or their biochemical activators (i.e., ROS, pathological protein aggregates, etc.) could represent a promising therapeutic strategy in order to prevent and ameliorate symptoms and the progression of neurodegenerative disorders.

## 4. Targeting Neuroinflammation and Oxidative Stress in Preclinical Models: Neuroprotective Role of Essential Oils

Derived from different parts of aromatic plants (leaf, flowers, seeds, roots and fruits), EOs are complex, heterogenous liquid mixtures, made up of a great number of volatile substances up to 400. Of note, EO heterogeneity is a function of producer species as well as environmental conditions. Plants of the same species can belong to a different “chemotype” according to the major component of their essential oils. Terpenes, isoprene-unit-derived biomolecules, are the most abundant class of compounds within EOs, and their modification allows the biosynthesis of different types of terpenoids. However, propenylphenols and allylphenols are also some other important classes of phytochemicals found in EOs.

Essential oils have captured the attention of researchers, drawing interest due to their unique properties and potential health benefits. These metabolites have demonstrated a multitude of advantageous properties, showcasing their diverse array of potential benefits as they can act as antioxidants, antimicrobials, anticancer, as well as anti-inflammatory and neuroprotective agents [72,73,74,75,76]. In this review, we provide a description of recent research and findings on the neuroprotective role of essential oils and their constituents extracted from different plant species, through the modulation of neuroinflammation and oxidative stress in the CNS (Table 1 and Table 2).

### 4.1. Pinus Halepensis EO

Essential oil of the conifer plant species *Pinus halepensis* has shown neuroprotective beneficial effects in preclinical models of Aβ-induced neurotoxicity. Postu et al. [77] studied the effects of EO from a Moroccan population of *Pinus halepensis*, in rats, following their exposure to Aβ. Interestingly, in the hippocampus of rats, *Pinus halepensis* EO showed an inhibitory capacity on acetylcholinesterase (AChE) activity, which is known to be associated with a decrease in Aβ aggregate formation [71,77]. Furthermore, this EO showed strong antioxidant properties associated with an increased activity of antioxidant enzymes such as catalase (CAT), superoxide dismutase (SOD) and glutathione peroxidase (GSH-PX), as well as increased levels of glutathione (GSH), which overall were able to counteract oxidative stress in rats treated with Aβ and to improve their memory deficits [77]. In another study, Postu et al. [9] revealed that in previously Aβ-treated rats, *Pinus halapensis* EO counteracted DNA fragmentation and ameliorated Aβ-induced neuroinflammation by decreasing levels of IL-1β mRNA in the brain, highlighting its potential therapeutical application. The anti-inflammatory and antioxidant properties of *Pinus halepensis* EO might be related to its major terpenoid components, notably β-caryophyllene, α-pinene and myrcene, which could activate different neuroprotective pathways such as CB2 signaling and the Nrf2/Keap1 axis [9,78]. Tian et al. [79] studied the effects of β-caryophyllene on ischemic stroke in mice. The results revealed that this sesquiterpene mediates neuroprotection through the downregulation of TLR4, which in turn stimulates the shift of microglia toward the M2 anti-inflammatory phenotype (as evidenced by upregulation of M2 signatures TGFβ, IL-10, CD206, ARG1 and downregulation of M1-related markers iNOS, TNFα, IL-1β) and the rescue of mice from neurologic deficits [79].

Askari et al. [80] explored the therapeutic efficacy of β-caryophyllene on experimental autoimmune encephalomyelitis mice (an MS in vivo model) [80]. This research highlights that this bioactive compound was able to ameliorate clinical manifestations in diseased mice through an increase in M2 microglia, Th2 and Treg immunosuppressive lymphocytes (along with a decrease in pro-inflammatory mediators), in a CB2-signaling-dependent manner [80]. Previous findings state that the β-caryophyellene stimulation of CB2 signaling can enhance PPARγ activity [81]. This receptor, ultimately, might regulate the neuroprotective phenotypic switch of microglia, as it inhibits p38, STAT1 and NF-κB pro-inflammatory pathways. Furthermore, PPARγ is an important regulator of microglia bioenergetics, as its activity is associated with a shift toward oxidative metabolism, which seems to be necessary in sustaining the functions of the neuroprotective state of microglia, while conversely, the pro-inflammatory phenotype relies upon glycolytic metabolism [82].

Azimullah et al. [83] studied the role of myrcene in ameliorating oxidative stress, neuroinflammation and α-synuclein clearance in a rodent model of PD induced by the pesticide rotenone. It was found that, in this PD animal model, myrcene (pre-treatment 30 min prior to rotenone injection) could increase autophagic flux and α-synuclein clearance, and then reduce microglia and astrocyte neurotoxic activation markers (GFAP and Iba1 overexpression) along with pro-inflammatory mediator release and ROS/RNS production. Collectively, myrcene was able to relieve neurodegeneration of the dopaminergic neurons in the substantia nigra pars compacta of PD rats [83].

### 4.2. Citrus Bergamia EO

*Citrus bergamia*, also known as “bergamot”, is a plant species belonging to the Rutaceae family that grows in Southern Italy and possesses diverse beneficial properties for human health [84]. Of importance, essential oil derived from bergamot showed neuroprotective properties.

In rat models of neurotoxicity induced by titanium dioxide nanoparticles or aluminum, bergamot EO co-treatment has been able to ameliorate oxidative damage and neuroinflammation in the hippocampus and frontal cortex regions [85,86]. Interestingly, EO induced an increase in antioxidant enzyme levels and a decrease in pro-inflammatory cytokine release (TNF-α, IL-1β, IL-6), lipid peroxidation and DNA damage [85,86].

The monoterpene limonene is one of the major components of bergamot EO and was found to represent up to 60% of the total amount [85,86]. In a recent paper, Eddin et al. [87] investigated the benefits of limonene consumption in rotenone-induced PD rats. In this study, limonene pre-treatment led to an increase in free radical scavenging capacities and a decrease in lipid peroxidation and pro-inflammatory cytokine release in the midbrain of rats. Interestingly, limonene also led to a decrease in Iba1-positive microglia and GFAP-positive astrocytes [87]. Remarkably, the upregulation of these proteins is associated with pro-inflammatory processes and a shift toward a neurotoxic phenotype [44,87]. In accordance, the inhibition of pro-inflammatory transcription factors and pathways such as NF-κB and MAPKs (JNK and p38) was evidenced after limonene exposure. Furthermore, this phytochemical enhanced the expression of BDNF (a neurotrophic factor associated with PD development when lacking) and the downregulation of αSyn. Altogether, in rats, limonene has demonstrated the ability to preserve dopaminergic neurons, shielding them against neurodegeneration [87].

### 4.3. Origanum vulgare EO

*Origanum vulgare* is a plant of the Mediterranean flora belonging to the Lamiaceae family with reported anti-inflammatory and repair-promoting beneficial properties [72].

Capatina et al. [8] investigated the neuroprotective attributes of Origanum vulgare spp. hirtum essential oil on a zebrafish model of scopolamine-induced neurotoxicity. EO pre-treatment was beneficial against oxidative stress and enhanced levels of antioxidant enzymes and glutathione, which ultimately led to a decrease in lipid peroxidation in the animal brain [8].

Additionally, Origanum vulgare EO reduced AChE activity and cholinergic deficits, which was associated with enhanced cognitive skills in zebrafish [8]. Of note, thymol and carvacrol (chemically represented by oxygenated monoterpenes) are two of the most abundant biomolecules that can be found in Origanum vulgare EO [8,72].

In recent research, thymol showed promising antioxidant and anti-inflammatory neuroprotective effects in rat brains, as well as excitotoxicity induced by elevated doses of monosodium glutamate. Particularly, thymol exerted its protective role through upregulation of the Nrf2/Heme Oxygenase (HO)-1 antioxidant pathway and downregulation of pro-inflammatory mediators such as TLR4, NLRP3 and NF-κB along with pro-inflammatory cytokines TNF-α and IL-1. Additionally, the observed neuroprotective outcomes were linked to a reduction in the expression of GFAP, the astroglial biomarker of activation following an injury [88].

In another study on thymol, Javed et al. [89] evidenced analogous results, where this terpenoid phenol protected a rotenone-induced rat model of PD (thymol was administered 30 min prior to rotenone injection) from neurodegeneration through its antioxidant and anti-inflammatory effects [89].

Likewise, carvacrol showed pronounced anti-inflammatory and antioxidant activities when administered after LPS injection in rats and rescued them from memory impairment [90]. In further research, carvacrol treatment after autoimmune encephalomyelitis induction in mice (representing a multiple sclerosis in vivo model) showed immunosuppressive effects as it decreased immune cell infiltrations in the spinal cord. Moreover, in these mice, carvacrol diminished pro-inflammatory cytokine release (IFN-γ, IL-6, IL-17) and augmented the secretion of the anti-inflammatory ones (TGFβ, IL-10) compared to untreated mice. Importantly, carvacrol treatment was associated with better remission in this MS in vivo model [91].

These results underscore the pivotal role of carvacrol and thymol in contributing to the beneficial properties of Origanum vulgare EO.

### 4.4. Rosmarinus Officinalis EO

A common plant of Mediterranean countries, Rosmarinus officinalis (family Lamiaceae), has been described as a plant with several positive biological properties, showing antioxidant, antimicrobial and anti-inflammatory activities [92,93]. In a zebrafish model of scopolamine-induced neurotoxicity, pre-treatment with Rosmarinus officinalis EO reduced AChE activity and increased antioxidant defenses in the brain, associated with enhanced cognitive functions [94]. Furthermore, Rosmarinus officinalis EO has been found to be enriched in eucalyptol (1,8-cineole), a terpenoid oxide [94]. In a rat model of early brain injury after subarachnoid hemorrhage, eucalyptol pre- and post-treatment ameliorated neuronal apoptosis and neurological deficits. Notably, it showed antioxidant characteristics, enhancing the Nrf2 pathway and ROS scavenging activity through SOD and GSH-Px. Additionally, eucalyptol inhibits NF-κB and pro-inflammatory microglia activation, which consequently led to a decrease in pro-inflammatory cytokine release [95].

Similarly, antioxidant and anti-inflammatory effects of eucalyptol have been evidenced in a rat model of hepatic encephalopathy induced by hyperammonemia injections (prior to eucalyptol treatment) [96]. Moreover, in an in vitro model of Alzheimer’s disease, eucalyptol pretreatment of the PC-12 cell line induced by Aβ led to a reduction in ROS, NO and pro-inflammatory markers such as COX-2, NOS-2, TNFα, IL-6, IL-1β and NF-κB compared to cells solely exposed to Aβ. Furthermore, in the same study, eucalyptol pretreatment restored the cell viability of PC-12 treated with Aβ, which might be a direct consequence of ROS scavenger activity of this bioactive phytomolecule [97].

### 4.5. Lavandula Augustifolia EO

Lavandula augustifolia (known as lavender) belongs to the Lamiaceae family and it is native to Mediterranean countries like Italy, Spain and France [98]. Lavandula spp., owing to their rich essential oil composition, finds versatile applications across different fields, ranging from medical (mostly used for anxiety and depression treatment) to cosmetic uses. Lavandula augustifolia EO showed antioxidant activity as it reduced cellular death in neuroblastoma cell line SH- SY5Y treated with hydrogen peroxide, especially at the longest incubation time (24 h) prior to the treatment with the toxicant [99]. Interestingly, Lavandula augustifolia EO, with its major components linalool and linalyl acetate, exhibits affinity for the NMDA receptor on the [^3^H]-CGP39653 binding assay [99]. In neuronal NGF-differentiated PC-12 cells, Lavandula augustifolia EO was neuroprotective against ROS induced by Aβ exposure [100]. The authors of this latter study hypothesize that this beneficial effect might be due to lavender EO’s ability to prevent Aβ-induced dysregulation of the intracellular influx of Ca^2+^ through the NMDA receptor, which in turn leads to ROS and RNS production and neuronal cell death [19,100].

In a rat model of dementia induced by scopolamine, the inhalation of lavender oil 30 min prior to toxicant induction significantly increased the antioxidant enzyme protein levels, and led to a decrease in lipid peroxidation and DNA fragmentation [101]. Furthermore, lavender oil pre-treatment counteracted the decline in neurogenesis induced by high doses of corticosterone in rats [102]. Of note, despite its well-recognized anti-inflammatory properties, elevated cortisol levels induce neuroinflammation and are additionally linked to cognitive decline and neurodegeneration. Therefore, counteracting high doses of cortisol may offer benefits in preventing and alleviating symptoms associated with brain disorders [103].

In BV-2 microglia cells, linalool pre-treatment reversed the LPS-induced increase in pro-inflammatory mediators by upregulating the Nrf2/HO-1 pathway [104].

In different in vivo AD mice models, notably APP_Swe_/PSEN_M146V_/MAPT_P301L_ triple transgenic (3XTg-AD) and Aβ-injected mice, linalool was beneficial against neurodegeneration and cognitive deficits through a decrease in neuroinflammation by the downregulation of pro-inflammatory cytokines and oxidative stress through Nrf2 upregulation [105,106].

### 4.6. Thymus Vulgaris EO

Thymus vulgaris is another species belonging to the Lamiaceae family, a native of Southern Europe that caught attention for its EO properties, particularly its antimicrobial, antifungal, antioxidant and anti-inflammatory activities [107].

Horvát et al. [108] tested three different chemotypes of Thymus vulgaris EO and their respective major compounds linalool, geraniol and thujanol, in BV-2 microglial cells treated with LPS. Interestingly, in this research, the decrease in pro-inflammatory cytokine (IL-6 and TNFα) mRNA and protein levels was stronger when EO or their major components alone were tested as pre-treatment before LPS stimulation than after LPS treatment or co-treatment. According to these results, the authors proposed that Thymus vulgaris EO or their singular components alone might be of better benefit in the prevention of neuroinflammation [108].

Another recent study using mice as a model investigated the role of Thymus vulgaris EO in reducing the chronic low-grade inflammation in various parts of the brain during aging (inflammaging), notably in the hippocampus, cerebral cortex and cerebellum. Intriguingly, inflammatory (IL-1β, IL-6) and aging markers (telomere length) showed a tendency for a reduction in chronologically aged mice fed with Thymus vulgaris EO compared with negative controls, and their survival rate was also higher [109]. These findings suggest that Thymus vulgaris EOs might be beneficial in preventing neurodegenerative diseases associated with neuroinflammation arising in the aging brain [109]. The neuroprotective roles of Thymus vulgaris EO might be attributed to its strong antioxidant capacities, as observed in a scopolamine-induced neurotoxicity in vivo model in zebrafish. EO treatment, prior to exposure to scopolamine, improved ROS scavenging defenses in the brain, which were correlated with enhanced cognitive functions in this experimental model [110].

Further research reported that the geraniol (one of the major compounds found in Thymus vulgaris EO, as mentioned above) pre-treatment of SK-N-SH cells exposed to rotenone (representing an in vitro model of PD) can improve the autophagic clearance of α-Syn and damaged mitochondria, which in turn positively affects proteostasis and mitigates oxidative stress [111]. Moreover, in this study, geraniol led to a reduction in the levels of endoplasmic reticulum (ER) stress sensors IRE1α, PERK and ATF6α [111]. Notably, prolonged ER stress is associated with neuroinflammation and neuronal apoptosis [112].

In addition, in vivo, geraniol improved neuroinflammation and oxidative stress, as well as cognitive skills, in mice fed with a high-fat diet prior to drug treatment [113].

Interestingly, Liu et al. [114] using a bioinformatic approach investigated geraniol targets, and the results revealed that geraniol potentially interacts with 29 AD-related targets. Remarkably, among these targets were JAK1 and JAK2, whose dysregulation is closely linked with neuroinflammation and brain cell survival [114].

### 4.7. Satureja Khuzistanica EO

Satureja khuzistanica (Lamiaceae family) is a herbal medicine endemic of Iran, well recognized for its antioxidant, antidiabetic, antiseptic and anti-inflammatory effects [115]. The potential neurotherapeutic utility of EO extracted from Satureja khuzistanica and its major component carvacrol has been widely studied in the context of neuroinflammation after traumatic brain injury (TBI) [116,117,118].

Satureja khuzistanica EO treatment demonstrated wide anti-inflammatory properties as it decreased the levels of IL-1β, NF-κB, IL-6 and TNFα, and increased IL-10 in the brain of rats 24 h after TBI. Accordingly, in TBI rats, EO reduced BBB permeability (evaluated with the Evans blue dye test), brain edema, leukocyte infiltration, cleaved caspase-3 (pro-apoptotic marker) and neuronal vacuolization (a morphological feature of neuronal damage). Along the same lines, the anti-inflammatory effects of Satureja khuzistanica EO led to an improvement in neurological impairments in treated rats after TBI, as they showed significantly higher veterinary comma scale scores than the untreated group [116,117].

Up to 90% of the total composition of Satureja khuzistanica EO is represented by carvacrol, and its molecular mechanism in regulating BBB permeability in TBI rats has been investigated. Of note, carvacrol administration reduces matrix metalloprotease-9 protein expression in brain rats after TBI induction, which in turn protects tight junction protein (ZO-1, occludin and claudin-5) from degradation and thus BBB integrity [118].

Furthermore, in another study, carvacrol rescued neurotoxicity induced by TBI in rats, through the downregulation of transient receptor potential melastatin 7 (TRPM7) [119]. In the context of TBI, an excessive divalent cation (Ca^2+^, Zn^2+^, Mg^2+^) influx through TRPM7 in neurons and microglia leads to ROS and RNS production and, in turn, neuronal death and M1 microglia activation. Thus, TRPM7 inhibition alleviates neuroinflammation and cell death in the brain after TBI in rats [119].

### 4.8. Jasminum Grandiflorum EO

Flower and leaf extracts of Jasminum grandiflorum (family Oleaceae) were reported to induce remarkable positive effects in alleviating oxidative stress and inflammation and enhancing wound healing in the context of hepatic injury and skin burn in mice [120,121].

In regard to neurodegenerative disorders, Lu et al. [122] explored the role of Jasminum grandiflorum EO in counteracting neuroinflammation and oxidative stress in the BV-2 cell line [122]. In particular, the LPS treatment of BV-2 microglial cells induced morphological changes typical of the activated pro-inflammatory state, such as enlarged soma and dendritic arbors, while co-treatment with EO could restore the quiescent morphology as in the negative control group (small soma and few pseudopodia), indicating that this volatile compound could mitigate the microglia inflammatory phenotype. According to these morphological changes, Jasminum grandiflorum led to a decrease in Iba-1 expression (a marker of microglia activation) along with a decrease in TNFα, IL-1β, ROS and NO. Interestingly, the best results were obtained with the lowest concentration (7.5 μg/mL) of EO used in this study [122].

Furthermore, employing an in silico approach, the authors made a prediction of the possible targets of the 34 volatile constituents identified in the mixture. Among a total of 346 predicted targets, 315 were found to be related to inflammatory and neuro-inflammatory processes. The protein–protein interaction network of these inflammatory-related targets revealed that the top five proteins with the highest degree value and therefore the most impacted were SRC, EGFR, VEGFA, HSP90AA1 and ESR1. Moreover, the network analysis of interactions among Jasminum grandiflorum EO compounds, predicted targets and inflammatory-related pathways indicate that α-hexylcinnamaldehyde, nerolidol, hexahydrofamesyl acetone, dodecanal and decanal were the top five key EO constituents in regulating inflammatory processes. Of relevance, the five predicted targets are all connected with the regulation of the TRP channel superfamily, which in turn is linked with the progression of neurodegenerative diseases [122].

To the best of our knowledge, up until today, there are no in vitro or in vivo studies that aimed to investigate the precise mechanisms of these EO components in modulating neuroinflammation through the abovementioned predicted targets; thus, these findings provide new insights for further experiments.

### 4.9. Acorus Tatarinowii EO

Acorus tatarinowii is a natural medicinal herb common in Asian regions, belonging to the family Acoraceae and abundant in phytochemicals with well-established pharmacological activities in the CNS. Its extracts, in particular, demonstrate antiepileptic, antianxiety, antidepressant, antifatigue and neuroprotective therapeutic effects [123].

Xu et al. [124] investigated the anti-neuroinflammatory molecular mechanisms underlying the improvement in cognitive impairments in 3XTg-AD mice treated with EO extracted from Acorus tatarinowii [124]. Acarus tatarinowii EO treatment reduced protein levels of AD hallmarks Aβ and phosphorylated (p)-Tau. Moreover, it relieved neuronal loss and injury morphological features in the AD-mice hippocampal tissues (i.e., disordered arrangement, shrunk and broken nucleus). The beneficial effects in counteracting neurodegeneration of this natural extract are associated with its anti-neuroinflammatory properties. The EO treatment of AD mice could strongly reduce the NLRP3-inflammasome signaling pathway and pyroptosis markers as indicated by reduced NLRP3, caspase-1, ASC and cleaved-gasdermin D at the mRNA and protein level. The amelioration of neuroinflammation in the brain of AD mice treated with EO was, in turn, beneficial in mitigating their cognitive impairments. In particular, EO treatment significantly reduced the time spent to find the hidden platform in the Morris water maze test and, furthermore, reduced the times mice stepped down from the platform onto the grid where the aversive stimulus was delivered and increased latency time in the step-down avoidance test [124].

Chemical composition analysis revealed that the most abundant constituent was asarone (α and β isomers, representing 70.08% and 4.43% of the total, respectively) [124].

Alpha-asarone is reported to exert therapeutical neuroprotective effects through different molecular pathways. Notably, it ameliorates dysmyelination due to mature oligodendrocyte loss after the induction of hypoxia-ischemia in neonatal rats, through the upregulation and activation of PPARγ in astrocytes, which in turn enhances glutamate transporter 1 expression and the clearance of excessive glutamate in the brain extracellular space, which might otherwise cause glutamate-mediated excitotoxicity in oligodendrocyte precursor cells, impeding their differentiation and inducing cell death [125]. In another study, α-asarone co-treatment reduced ROS levels, ER stress markers such as p-PERK, and, in turn, neuronal cell death induced by L-glutamate and tunicamycin (ER stress inducer) in mouse hippocampal HT-22 cells [126]. Interestingly, p-PERK activity can induce neuroinflammation through an atypical STING immune pathway in neurons, which sequentially leads to IFNβ release and microglia M1 activation through STAT1 [127].

After spinal cord injury (SCI) induction in rats, the oral administration of α-asarone (for 14 days) was able to ameliorate locomotor deficits through its marked anti-neuroinflammatory effects which could counteract secondary injury due to the inflammatory response [128]. In an injured spinal cord, α-asarone determined the shift of macrophages toward the anti-inflammatory and repair-promoting phenotype, as indicated by the reduced expression levels of pro-inflammatory mediators (TNF-α, IL-1β, IL-6 and chemokines such as monocyte chemoattractant protein 1 (MCP-1) and macrophage inflammatory protein 2 (MIP-2)), along with increased M2 markers (iNOS, Arg1, IL-4, IL-10). Moreover, α-asarone treatment led to a decrease in GFAP expression (indicative of astrocyte activation) and ameliorated glial scar formation by reactive astrocytes in the spinal cord [128].

Beta-asarone represents the cis isomer of asarone. Recent findings suggest that this phenylpropanoid can regulate different molecular mechanisms to retrieve axonal regeneration and apoptosis in mouse primary cortical neurons after scratch injury damage [129]. In detail, β-asarone can inhibit JNK-dependent c-jun phosphorylation and activation, which ultimately is unable to interact with the promoter region of the TNF-α gene to stimulate its transcription. Moreover, β-asarone induces the upregulation of UHFR1 protein that, in turn, recruits DNA methyltransferase 1 to induce TNF-α promoter methylation and thus gene expression silencing [129].

In the Aβ-stimulated PC-12 cell line, post-treatment with β-asarone reduced cellular cytotoxicity and annexin V-propidium iodide-positive apoptotic cells through its remarkable antioxidant properties. Of note, Aβ treatment increased lipid peroxidation and reduced CAT, SOD, GSH-PX and HO-1 antioxidant enzyme levels in PC12 cells, while post-treatment with β-asarone could restore antioxidant cellular defenses and retrieve oxidative stress induced by Aβ in a dose-dependent manner [130]. Further investigation of the molecular pathways implied in the antioxidant effects of β-asarone evidenced that this bioactive natural compound could upregulate the PI3K/Akt/Nrf2 antioxidant axis and therefore the transcription of antioxidant genes [130].

In a recent analysis using the network pharmacology computational approach and molecular docking, Ning et al. [131] individuated RELA, a subunit of the NF-κB complex, as a possible target of β-asarone [131]. Interestingly, at the highest dosage, β-asarone could significantly reduce RELA mRNA expression in a vascular dementia mouse model [131].

**Table 1:** Summary of essential oil effects in preclinical models of neuroinflammation and oxidative stress in the brain.

Essential Oil (EO)	Major Constituent	Preclinical Model	EO Preparation	Effect	References
*Pinus halepensis*	α-pynene, myrcene, β-caryophyllene	Aβ-induced AD in rats	1% Tween 80 solution	AChE inhibitor, antioxidant, anti-inflammatory, DNA fragmentation protector, nootropic	[9,77]
*Citrus bergamia*	limonene	Titanium dioxide- or aluminum-induced neurotoxicity in rats	soybean oil	Antioxidant, anti- inflammatory	[85,86]
*Origanum vulgare*	thymol, carvacrol	Scopolamine-induced neurotoxicity in zebrafish	1% Tween 80 solution	Antioxidant, nootropic, AChE inhibitor	[8]
*Rosmarinus officinalis*	eucalyptol	Scopolamine-induced neurotoxicity in zebrafish	n.a.	Antioxidant, AChE inhibitor, nootropic	[94]
*Lavandula angustifolia*	linalool	H_2_O_2_-treated SH-SY5Y cells, Aβ-treated NGF- differentiated PC-12 cells, Scopolamine-induced dementia in rats, Corticosterone-treated rats	1% Tween 80 or 20 solution	Antioxidant, NMDA receptor inhibitor, DNA fragmentation protector, neurogenesis promoter	[99,100,101]
*Thymus vulgaris*	linalool, geraniol, thujanol	LPS-treated BV-2 cells Chronologically aged mice Scopolamine-induced neurotoxicity in zebrafish	DMSO or 1% Tween 80 solution	Anti-inflammatory, decrease brain inflammaging, antioxidant, nootropic, AChE inhibitor	[108,109,110]
*Satureja khuzistanica*	carvacrol	Traumatic brain injury in rats	1% Tween 20	Anti-inflammatory, anti-apoptotic	[116,117,118]
*Jasminum grandiflorum*	α-hexylcinnamaldehyde nerolidol, hexahydrofarnesyl acetone, decanal, dodecanal (in silico-predicted key compounds in targeting neuroinflammation)	BV-2 microglial cell line	n.a.	Anti-inflammatory, antioxidant	[122]
*Acorus tatarinowii*	β-Asarone, α-Asarone	APP_Swe_/PSEN_M146V_/MAPT_P301L_ triple transgenic mice	n.a.	Anti-inflammatory (NRLP3-inflammasome inhibition), nootropic	[124]

n.a.: not available.

**Table 2:** Summary of molecular mechanisms of essential oils’ major constituents in counteracting neuroinflammation and oxidative stress in the brain.

EO Major Constituent	Chemical Structure	Molecular Mechanism	Experimental Model	Drug Preparation	References
β-caryophyllene	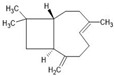	↑Nrf2 ↑CB2 ↑TGFβ ↑IL-10 ↑Arg1 ↑CD206 ↑SOD ↑CAT ↑GSH ↓TLR4 ↓iNOS ↓TNF-α ↓IL-1β ↓PGE_2_	Ischemic stroke in mice, Experimental autoimmune encephalomyelitis mice (multiple sclerosis in vivo model)	Dissolved in olive or corn oil	[9,79,80]
Myrcene	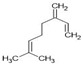	↑Nrf2/Keap1 ↑Autophagy ↑GSH ↑SOD ↑CAT ↓Iba1 (activated microglia) ↓GFAP (activated astrocytes) ↓iNOS ↓COX-2 ↓TNF-α ↓IL-1β ↓IL-6 ↓MMP-9	Rotenone-induced PD in rats	Dissolved in olive oil	[9,83]
Limonene	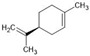	↑BDNF ↑GSH ↑SOD ↑CAT ↓NF-κB ↓p38 ↓JNK ↓α-Syn ↓Iba1 (activated microglia) ↓GFAP (activated astrocytes) ↓iNOS ↓COX-2 ↓TNF-α ↓IL-1β ↓IL-6	Rotenone-induced PD in rats	Dissolved in olive oil	[87]
Thymol	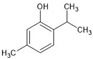	↑Nrf2/HO-1 ↑SOD ↑GSH ↑CAT ↓TLR4 ↓NLRP3 ↓NF-κB ↓IL-1 ↓TNFα ↓GFAP (activated astrocytes) ↓IL-6 ↓COX-2 ↓iNOS	Glutamate-induced excitotoxicity in rats, Rotenone-induced PD in rats	Dissolved in sunflower oil	[88,89]
Carvacrol	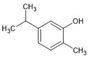	↑TGF-β ↑IL-10 ↑BDNF ↑SOD ↑BBB integrity ↓IFN-γ ↓IL-6 ↓IL-17 ↓NF-κB ↓TLR4 ↓iNOS ↓COX-2 ↓MMP-9 ↓TRPM7	LPS-treated rats, Experimental autoimmune encephalomyelitis, Traumatic brain injury in rats	Dissolved in 0.9% saline solution or 2% Tween 80 or 0.1% DMSO	[90,91,117,118,119]
Eucalyptol	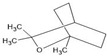	↑Nrf2 ↑SOD ↑GSH-Px ↓NF-κB ↓COX-2 ↓NOS-2 ↓TNFα ↓IL-6 ↓IL-1	Brain injury after subarachnoid hemorrhage in mice, Hyperammonemic rats, Aβ-toxicated PC-12 cells	Dissolved in corn oil	[95,96,97]
Linalool	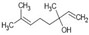	↑Nrf2/HO-1 ↓NMDA ↓PGE2 ↓NF-κB ↓TNFα ↓IL1β	PC-12 cells treated with Aβ, LPS-induced BV-2, Triple transgenic and Aβ-induced AD mice	Dissolved in PBS or saline solution with 2% Tween 80 and 1% DMSO	[100,104,105,106]
Geraniol	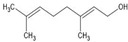	↑Autophagy ↑GSH ↑SOD ↓IL-6 ↓TNFα ↓α-Syn ↓PERK ↓IRE1α ↓ATF6α JAK1/2	Rotenone-toxicated SK-N-SH, Mice fed with high fat diet In silico prediction	Dissolved in saline solution	[111,113,114]
α-Hexylcinnamaldehyde	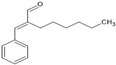	SRC, VEGFA, EGFR, HSP90AA1, ESR1	I In silico-predicted targets; Docking binding energies ≤ −3.9 kJ/mol	n.a.	[122]
Nerolidol	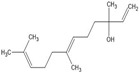				
Hexahydrofarnesyl acetone	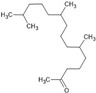				
Decanal	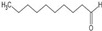				
Dodecanal	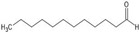				
α-Asarone	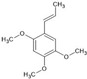	↑PPARγ-Glutamate transporter 1 ↑IL-10 ↑IL-4 ↑Arg1 ↓p-PERK (ER stress) ↓IL-6 ↓TNFα ↓IL-1β ↓iNOS ↓GFAP ↓MCP1 ↓MIP2	Hypoxia-ischemia neonatal rats, HT-22 cells, Spinal cord injury in rats	Dissolved in 0.5% carboxymethylcellulose	[125,126,128]
β-Asarone	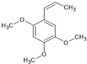	↑PI3K/Akt/Nrf2 ↑HO-1 ↑SOD ↑CAT ↑GSH-Px ↓TNFα (promoter DNA methylation) ↓JNK/c-JUN ↓RELA (NF-κB subunit)	Scratch-injured primary cortical mice neurons, Aβ-treated PC-12 cells, Vascular dementia mice	Dissolved in DMSO or 0.9% saline solution	[129,130,131]

↑: increase; ↓: decrease; n.a.: not available.

## 5. Conclusions

Neuroinflammation and oxidative stress are nowadays considered hallmarks of various brain disorders, including neurodegenerative diseases. However, there is still a great need to understand processes implicated in the development and evolution of progressive neurological conditions as well as an ongoing request for new effective drugs for their treatment. Despite their valuable contribution to the management of many brain diseases, available pharmacological therapies are not, in every case, efficacious and they show several side-effects.

In this regard, plant biomolecules have always attracted researchers for their health-promoting properties and indeed they represent a great source of new drugs. Essential oils showed promising results in many in vitro and in vivo preclinical models of neurodegenerative disorders, as they counteract oxidative stress and neuroinflammation and rescue from neuronal death and neurodegeneration, which ultimately lead to an improvement in disease-related symptoms. Nevertheless, precise mechanisms by which these oily mixtures exert their neuroprotective functions are still not fully elucidated, and even though a great number of studies focused on the role of singular components of essential oils, the understanding of their synergies is still lacking. Furthermore, regarding the essential oils cited in this paper, clinical trials evaluating the potential benefits of patients from these alternative therapies are still required. Of note, until today, even if with auspicious results in enhancing cognitive functions, only a few studies evaluated the potential advantages of essential oil usage in human in vivo models for neurodegenerative disease treatment; therefore, more clinical trials might be expected to occur in the future to clarify their possible utility in the management of these pathologies. In any case, this review aimed to summarize the most updated literature regarding the essential oils that have been most studied for application in pathologies associated with neuroinflammation.

## Data Availability

Data are contained within the article.

## References

[1] Kwon H.S., Koh S.H. (2020). Neuroinflammation in Neurodegenerative Disorders: The Roles of Microglia and Astrocytes. Transl. Neurodegener..

[2] Lyman M., Lloyd D.G., Ji X., Vizcaychipi M.P., Ma D. (2014). Neuroinflammation: The Role and Consequences. Neurosci. Res..

[3] Subhramanyam C.S., Wang C., Hu Q., Dheen S.T. (2019). Microglia-Mediated Neuroinflammation in Neurodegenerative Diseases. Semin. Cell Dev. Biol..

[4] Palpagama T.H., Waldvogel H.J., Faull R.L.M., Kwakowsky A. (2019). The Role of Microglia and Astrocytes in Huntington’s Disease. Front. Mol. Neurosci..

[5] Ransohoff R.M. (2016). How Neuroinflammation Contributes to Neurodegeneration. Science.

[6] Fanaro G.B., Marques M.R., Calaza K.d.C., Brito R., Pessoni A.M., Mendonça H.R., Lemos D.E.d.A., de Brito Alves J.L., de Souza E.L., Cavalcanti Neto M.P. (2023). New Insights on Dietary Polyphenols for the Management of Oxidative Stress and Neuroinflammation in Diabetic Retinopathy. Antioxidants.

[7] Rahimifard M., Maqbool F., Moeini-Nodeh S., Niaz K., Abdollahi M., Braidy N., Nabavi S.M., Nabavi S.F. (2017). Targeting the TLR4 Signaling Pathway by Polyphenols: A Novel Therapeutic Strategy for Neuroinflammation. Ageing Res. Rev..

[8] Capatina L., Napoli E.M., Ruberto G., Hritcu L. (2021). *Origanum vulgare* ssp. Hirtum (Lamiaceae) Essential Oil Prevents Behavioral and Oxidative Stress Changes in the Scopolamine Zebrafish Model. Molecules.

[9] Postu P.A., Mihasan M., Gorgan D.L., Sadiki F.Z., El Idrissi M., Hritcu L. (2022). Pinus Halepensis Essential Oil Ameliorates Aβ1-42-Induced Brain Injury by Diminishing Anxiety, Oxidative Stress, and Neuroinflammation in Rats. Biomedicines.

[10] Feng J., Wang J.-X., Du Y.-H., Liu Y., Zhang W., Chen J.-F., Liu Y.-J., Zheng M., Wang K.-J., He G.-Q. (2018). Dihydromyricetin Inhibits Microglial Activation and Neuroinflammation by Suppressing NLRP3 Inflammasome Activation in APP/PS1 Transgenic Mice. CNS Neurosci. Ther..

[11] Liu P., Zhou Y., Shi J., Wang F., Yang X., Zheng X., Wang Y., He Y., Xie X., Pang X. (2023). Myricetin Improves Pathological Changes in 3×Tg-AD Mice by Regulating the Mitochondria-NLRP3 Inflammasome-Microglia Channel by Targeting P38 MAPK Signaling Pathway. Phytomedicine.

[12] Guo S., Wang H., Yin Y. (2022). Microglia Polarization from M1 to M2 in Neurodegenerative Diseases. Front. Aging Neurosci..

[13] Liu W., Wang Y., Gong F., Rong Y., Luo Y., Tang P., Zhou Z., Zhou Z., Xu T., Jiang T. (2019). Exosomes Derived from Bone Mesenchymal Stem Cells Repair Traumatic Spinal Cord Injury by Suppressing the Activation of A1 Neurotoxic Reactive Astrocytes. J. Neurotrauma.

[14] Bordt E.A., Polster B.M. (2014). NADPH Oxidase- and Mitochondria-Derived Reactive Oxygen Species in Proinflammatory Microglial Activation: A Bipartisan Affair?. Free Radic. Biol. Med..

[15] Block M.L., Zecca L., Hong J.-S. (2007). Microglia-Mediated Neurotoxicity: Uncovering the Molecular Mechanisms. Nat. Rev. Neurosci..

[16] Mosley R.L., Benner E.J., Kadiu I., Thomas M., Boska M.D., Hasan K., Laurie C., Gendelman H.E. (2006). Neuroinflammation, Oxidative Stress and the Pathogenesis of Parkinson’s Disease. Clin. Neurosci. Res..

[17] Gaschler M.M., Stockwell B.R. (2017). Lipid Peroxidation in Cell Death. Biochem. Biophys. Res. Commun..

[18] Takeuchi H., Jin S., Wang J., Zhang G., Kawanokuchi J., Kuno R., Sonobe Y., Mizuno T., Suzumura A. (2006). Tumor Necrosis Factor-Alpha Induces Neurotoxicity via Glutamate Release from Hemichannels of Activated Microglia in an Autocrine Manner. J. Biol. Chem..

[19] Plotegher N., Filadi R., Pizzo P., Duchen M.R. (2021). Excitotoxicity Revisited: Mitochondria on the Verge of a Nervous Breakdown. Trends Neurosci..

[20] Leri M., Vasarri M., Carnemolla F., Oriente F., Cabaro S., Stio M., Degl’Innocenti D., Stefani M., Bucciantini M. (2023). EVOO Polyphenols Exert Anti-Inflammatory Effects on the Microglia Cell through TREM2 Signaling Pathway. Pharmaceuticals.

[21] Blevins H.M., Xu Y., Biby S., Zhang S. (2022). The NLRP3 Inflammasome Pathway: A Review of Mechanisms and Inhibitors for the Treatment of Inflammatory Diseases. Front. Aging Neurosci..

[22] Chen W., Guo C., Huang S., Jia Z., Wang J., Zhong J., Ge H., Yuan J., Chen T., Liu X. (2020). MitoQ Attenuates Brain Damage by Polarizing Microglia towards the M2 Phenotype through Inhibition of the NLRP3 Inflammasome after ICH. Pharmacol. Res..

[23] Wang S., Yuan Y.-H., Chen N.-H., Wang H.-B. (2019). The Mechanisms of NLRP3 Inflammasome/Pyroptosis Activation and Their Role in Parkinson’s Disease. Int. Immunopharmacol..

[24] Kann O., Almouhanna F., Chausse B. (2022). Interferon γ: A Master Cytokine in Microglia-Mediated Neural Network Dysfunction and Neurodegeneration. Trends Neurosci..

[25] Rock R.B., Hu S., Deshpande A., Munir S., May B.J., Baker C.A., Peterson P.K., Kapur V. (2005). Transcriptional Response of Human Microglial Cells to Interferon-Gamma. Genes. Immun..

[26] Yao Y.-Y., Li R., Guo Y.-J., Zhao Y., Guo J.-Z., Ai Q.-L., Zhong L.-M., Lu D. (2022). Gastrodin Attenuates Lipopolysaccharide-Induced Inflammatory Response and Migration via the Notch-1 Signaling Pathway in Activated Microglia. Neuromol. Med..

[27] Zhang Y.-H., Wang T., Li Y.-F., Deng Y.-N., Shen F.-G. (2023). Roles of the Notch Signaling Pathway and Microglia in Autism. Behav. Brain Res..

[28] Capiralla H., Vingtdeux V., Zhao H., Sankowski R., Al-Abed Y., Davies P., Marambaud P. (2012). Resveratrol Mitigates Lipopolysaccharide- and Aβ-Mediated Microglial Inflammation by Inhibiting the TLR4/NF-κB/STAT Signaling Cascade. J. Neurochem..

[29] Cui W., Sun C., Ma Y., Wang S., Wang X., Zhang Y. (2020). Inhibition of TLR4 Induces M2 Microglial Polarization and Provides Neuroprotection via the NLRP3 Inflammasome in Alzheimer’s Disease. Front. Neurosci..

[30] Hu X., Leak R.K., Shi Y., Suenaga J., Gao Y., Zheng P., Chen J. (2015). Microglial and Macrophage Polarization—New Prospects for Brain Repair. Nat. Rev. Neurol..

[31] Zhang J.-M., An J. (2007). Cytokines, Inflammation, and Pain. Int. Anesthesiol. Clin..

[32] Li Z., Wang L., Ren Y., Huang Y., Liu W., Lv Z., Qian L., Yu Y., Xiong Y. (2022). Arginase: Shedding Light on the Mechanisms and Opportunities in Cardiovascular Diseases. Cell Death Discov..

[33] Tang Y., Li T., Li J., Yang J., Liu H., Zhang X.J., Le W. (2014). Jmjd3 Is Essential for the Epigenetic Modulation of Microglia Phenotypes in the Immune Pathogenesis of Parkinson’s Disease. Cell Death Differ..

[34] Gazi U., Martinez-Pomares L. (2009). Influence of the Mannose Receptor in Host Immune Responses. Immunobiology.

[35] Rahimian R., Belliveau C., Chen R., Mechawar N. (2022). Microglial Inflammatory-Metabolic Pathways and Their Potential Therapeutic Implication in Major Depressive Disorder. Front. Psychiatry.

[36] He Y., Gao Y., Zhang Q., Zhou G., Cao F., Yao S. (2020). IL-4 Switches Microglia/Macrophage M1/M2 Polarization and Alleviates Neurological Damage by Modulating the JAK1/STAT6 Pathway Following ICH. Neuroscience.

[37] Wang Y., Lin Y., Wang L., Zhan H., Luo X., Zeng Y., Wu W., Zhang X., Wang F. (2020). TREM2 Ameliorates Neuroinflammatory Response and Cognitive Impairment via PI3K/AKT/FoxO3a Signaling Pathway in Alzheimer’s Disease Mice. Aging.

[38] Zhang J., Zheng Y., Luo Y., Du Y., Zhang X., Fu J. (2019). Curcumin Inhibits LPS-Induced Neuroinflammation by Promoting Microglial M2 Polarization via TREM2/ TLR4/ NF-κB Pathways in BV2 Cells. Mol. Immunol..

[39] Liu W., Rong Y., Wang J., Zhou Z., Ge X., Ji C., Jiang D., Gong F., Li L., Chen J. (2020). Exosome-Shuttled miR-216a-5p from Hypoxic Preconditioned Mesenchymal Stem Cells Repair Traumatic Spinal Cord Injury by Shifting Microglial M1/M2 Polarization. J. Neuroinflamm..

[40] Vergadi E., Ieronymaki E., Lyroni K., Vaporidi K., Tsatsanis C. (2017). Akt Signaling Pathway in Macrophage Activation and M1/M2 Polarization. J. Immunol..

[41] Bellot-Saez A., Stevenson R., Kékesi O., Samokhina E., Ben-Abu Y., Morley J.W., Buskila Y. (2021). Neuromodulation of Astrocytic K+ Clearance. Int. J. Mol. Sci..

[42] Gomolka R.S., Hablitz L.M., Mestre H., Giannetto M., Du T., Hauglund N.L., Xie L., Peng W., Martinez P.M., Nedergaard M. (2023). Loss of Aquaporin-4 Results in Glymphatic System Dysfunction via Brain-Wide Interstitial Fluid Stagnation. eLife.

[43] Phatnani H., Maniatis T. (2015). Astrocytes in Neurodegenerative Disease. Cold Spring Harb. Perspect. Biol..

[44] Liddelow S.A., Guttenplan K.A., Clarke L.E., Bennett F.C., Bohlen C.J., Schirmer L., Bennett M.L., Münch A.E., Chung W.-S., Peterson T.C. (2017). Neurotoxic Reactive Astrocytes Are Induced by Activated Microglia. Nature.

[45] Liu L.-R., Liu J.-C., Bao J.-S., Bai Q.-Q., Wang G.-Q. (2020). Interaction of Microglia and Astrocytes in the Neurovascular Unit. Front. Immunol..

[46] Xie L., Zhang N., Zhang Q., Li C., Sandhu A.F., Iii G.W., Lin S., Lv P., Liu Y., Wu Q. (2020). Inflammatory Factors and Amyloid β-Induced Microglial Polarization Promote Inflammatory Crosstalk with Astrocytes. Aging.

[47] Li S., Fang Y., Zhang Y., Song M., Zhang X., Ding X., Yao H., Chen M., Sun Y., Ding J. (2022). Microglial NLRP3 Inflammasome Activates Neurotoxic Astrocytes in Depression-like Mice. Cell Rep..

[48] Xiao T., Ji H., Shangguan X., Qu S., Cui Y., Xu J. (2022). NLRP3 Inflammasome of Microglia Promotes A1 Astrocyte Transformation, Neo-Neuron Decline and Cognition Impairment in Endotoxemia. Biochem. Biophys. Res. Commun..

[49] Sofroniew M.V. (2009). Molecular Dissection of Reactive Astrogliosis and Glial Scar Formation. Trends Neurosci..

[50] Wang H., Song G., Chuang H., Chiu C., Abdelmaksoud A., Ye Y., Zhao L. (2018). Portrait of Glial Scar in Neurological Diseases. Int. J. Immunopathol. Pharmacol..

[51] Hamby M.E., Sofroniew M.V. (2010). Reactive Astrocytes as Therapeutic Targets for CNS Disorders. Neurotherapeutics.

[52] Qian D., Li L., Rong Y., Liu W., Wang Q., Zhou Z., Gu C., Huang Y., Zhao X., Chen J. (2019). Blocking Notch Signal Pathway Suppresses the Activation of Neurotoxic A1 Astrocytes after Spinal Cord Injury. Cell Cycle.

[53] Wu M., Wang L., Li F., Hu R., Ma J., Zhang K., Cheng X. (2020). Resveratrol Downregulates STAT3 Expression and Astrocyte Activation in Primary Astrocyte Cultures of Rat. Neurochem. Res..

[54] Chang J., Qian Z., Wang B., Cao J., Zhang S., Jiang F., Kong R., Yu X., Cao X., Yang L. (2023). Transplantation of A2 Type Astrocytes Promotes Neural Repair and Remyelination after Spinal Cord Injury. Cell Commun. Signal.

[55] Li T., Liu T., Chen X., Li L., Feng M., Zhang Y., Wan L., Zhang C., Yao W. (2020). Microglia Induce the Transformation of A1/A2 Reactive Astrocytes via the CXCR7/PI3K/Akt Pathway in Chronic Post-Surgical Pain. J. Neuroinflamm..

[56] Neal M., Luo J., Harischandra D.S., Gordon R., Sarkar S., Jin H., Anantharam V., Désaubry L., Kanthasamy A., Kanthasamy A. (2018). Prokineticin-2 Promotes Chemotaxis and Alternative A2 Reactivity of Astrocytes. Glia.

[57] Stephenson J., Nutma E., van der Valk P., Amor S. (2018). Inflammation in CNS Neurodegenerative Diseases. Immunology.

[58] Guo J., Huang X., Dou L., Yan M., Shen T., Tang W., Li J. (2022). Aging and Aging-Related Diseases: From Molecular Mechanisms to Interventions and Treatments. Signal Transduct. Target. Ther..

[59] Scheiblich H., Trombly M., Ramirez A., Heneka M.T. (2020). Neuroimmune Connections in Aging and Neurodegenerative Diseases. Trends Immunol..

[60] Li M.D., Burns T.C., Morgan A.A., Khatri P. (2014). Integrated Multi-Cohort Transcriptional Meta-Analysis of Neurodegenerative Diseases. Acta Neuropathol. Commun..

[61] Ross C.A., Poirier M.A. (2004). Protein Aggregation and Neurodegenerative Disease. Nat. Med..

[62] Hinkle J.T., Patel J., Panicker N., Karuppagounder S.S., Biswas D., Belingon B., Chen R., Brahmachari S., Pletnikova O., Troncoso J.C. (2022). STING Mediates Neurodegeneration and Neuroinflammation in Nigrostriatal α-Synucleinopathy. Proc. Natl. Acad. Sci. USA.

[63] Gulen M.F., Samson N., Keller A., Schwabenland M., Liu C., Glück S., Thacker V.V., Favre L., Mangeat B., Kroese L.J. (2023). cGAS-STING Drives Ageing-Related Inflammation and Neurodegeneration. Nature.

[64] Gordon R., Albornoz E.A., Christie D.C., Langley M.R., Kumar V., Mantovani S., Robertson A.A.B., Butler M.S., Rowe D.B., O’Neill L.A. (2018). Inflammasome Inhibition Prevents α-Synuclein Pathology and Dopaminergic Neurodegeneration in Mice. Sci. Transl. Med..

[65] Lee E., Hwang I., Park S., Hong S., Hwang B., Cho Y., Son J., Yu J.-W. (2019). MPTP-Driven NLRP3 Inflammasome Activation in Microglia Plays a Central Role in Dopaminergic Neurodegeneration. Cell Death Differ..

[66] Liu Y., Dai Y., Li Q., Chen C., Chen H., Song Y., Hua F., Zhang Z. (2020). Beta-Amyloid Activates NLRP3 Inflammasome via TLR4 in Mouse Microglia. Neurosci. Lett..

[67] Jung E.S., Suh K., Han J., Kim H., Kang H.-S., Choi W.-S., Mook-Jung I. (2022). Amyloid-β Activates NLRP3 Inflammasomes by Affecting Microglial Immunometabolism through the Syk-AMPK Pathway. Aging Cell.

[68] Moonen S., Koper M.J., Van Schoor E., Schaeverbeke J.M., Vandenberghe R., von Arnim C.A.F., Tousseyn T., De Strooper B., Thal D.R. (2023). Pyroptosis in Alzheimer’s Disease: Cell Type-Specific Activation in Microglia, Astrocytes and Neurons. Acta Neuropathol..

[69] McKenzie B.A., Mamik M.K., Saito L.B., Boghozian R., Monaco M.C., Major E.O., Lu J.-Q., Branton W.G., Power C. (2018). Caspase-1 Inhibition Prevents Glial Inflammasome Activation and Pyroptosis in Models of Multiple Sclerosis. Proc. Natl. Acad. Sci. USA.

[70] Van Schoor E., Ospitalieri S., Moonen S., Tomé S.O., Ronisz A., Ok O., Weishaupt J., Ludolph A.C., Van Damme P., Van Den Bosch L. (2022). Increased Pyroptosis Activation in White Matter Microglia Is Associated with Neuronal Loss in ALS Motor Cortex. Acta Neuropathol..

[71] Bartolini M., Bertucci C., Cavrini V., Andrisano V. (2003). Beta-Amyloid Aggregation Induced by Human Acetylcholinesterase: Inhibition Studies. Biochem. Pharmacol..

[72] Avola R., Granata G., Geraci C., Napoli E., Graziano A.C.E., Cardile V. (2020). Oregano (*Origanum vulgare* L.) Essential Oil Provides Anti-Inflammatory Activity and Facilitates Wound Healing in a Human Keratinocytes Cell Model. Food Chem. Toxicol..

[73] de Lavor É.M., Fernandes A.W.C., de Andrade Teles R.B., Leal A.E.B.P., de Oliveira Júnior R.G., Gama E Silva M., de Oliveira A.P., Silva J.C., de Moura Fontes Araújo M.T., Coutinho H.D.M. (2018). Essential Oils and Their Major Compounds in the Treatment of Chronic Inflammation: A Review of Antioxidant Potential in Preclinical Studies and Molecular Mechanisms. Oxid. Med. Cell. Longev..

[74] Mladenović M., Astolfi R., Tomašević N., Matić S., Božović M., Sapienza F., Ragno R. (2023). In Vitro Antioxidant and In Vivo Antigenotoxic Features of a Series of 61 Essential Oils and Quantitative Composition-Activity Relationships Modeled through Machine Learning Algorithms. Antioxidants.

[75] Ramsey J.T., Shropshire B.C., Nagy T.R., Chambers K.D., Li Y., Korach K.S. (2020). Essential Oils and Health. Yale J. Biol. Med..

[76] Russo A., Bruno M., Avola R., Cardile V., Rigano D. (2020). Chamazulene-Rich Artemisia Arborescens Essential Oils Affect the Cell Growth of Human Melanoma Cells. Plants.

[77] Postu P.A., Sadiki F.Z., El Idrissi M., Cioanca O., Trifan A., Hancianu M., Hritcu L. (2019). Pinus Halepensis Essential Oil Attenuates the Toxic Alzheimer’s Amyloid Beta (1-42)-Induced Memory Impairment and Oxidative Stress in the Rat Hippocampus. Biomed. Pharmacother..

[78] Irrera N., D’Ascola A., Pallio G., Bitto A., Mannino F., Arcoraci V., Rottura M., Ieni A., Minutoli L., Metro D. (2020). β-Caryophyllene Inhibits Cell Proliferation through a Direct Modulation of CB2 Receptors in Glioblastoma Cells. Cancers.

[79] Tian X., Liu H., Xiang F., Xu L., Dong Z. (2019). β-Caryophyllene Protects against Ischemic Stroke by Promoting Polarization of Microglia toward M2 Phenotype via the TLR4 Pathway. Life Sci..

[80] Askari V.R., Baradaran Rahimi V., Shafiee-Nick R. (2023). Low Doses of β-Caryophyllene Reduced Clinical and Paraclinical Parameters of an Autoimmune Animal Model of Multiple Sclerosis: Investigating the Role of CB2 Receptors in Inflammation by Lymphocytes and Microglial. Brain Sci..

[81] Youssef D.A., El-Fayoumi H.M., Mahmoud M.F. (2019). Beta-Caryophyllene Alleviates Diet-Induced Neurobehavioral Changes in Rats: The Role of CB2 and PPAR-γ Receptors. Biomed. Pharmacother..

[82] Fumagalli M., Lombardi M., Gressens P., Verderio C. (2018). How to Reprogram Microglia toward Beneficial Functions. Glia.

[83] Azimullah S., Jayaraj R.L., Meeran M.F.N., Jalal F.Y., Adem A., Ojha S., Beiram R. (2023). Myrcene Salvages Rotenone-Induced Loss of Dopaminergic Neurons by Inhibiting Oxidative Stress, Inflammation, Apoptosis, and Autophagy. Molecules.

[84] Nauman M.C., Johnson J.J. (2019). Clinical Application of Bergamot (*Citrus bergamia*) for Reducing High Cholesterol and Cardiovascular Disease Markers. Integr. Food Nutr. Metab..

[85] Cui Y., Che Y., Wang H. (2020). Bergamot Essential Oil Attenuate Aluminum-Induced Anxiety-like Behavior through Antioxidation, Anti-Inflammatory and GABA Regulation in Rats. Food Chem. Toxicol..

[86] Cui Y., Che Y., Wang H. (2021). Nono-Titanium Dioxide Exposure during the Adolescent Period Induces Neurotoxicities in Rats: Ameliorative Potential of Bergamot Essential Oil. Brain Behav..

[87] Eddin L.B., Azimullah S., Jha N.K., Nagoor Meeran M.F., Beiram R., Ojha S. (2023). Limonene, a Monoterpene, Mitigates Rotenone-Induced Dopaminergic Neurodegeneration by Modulating Neuroinflammation, Hippo Signaling and Apoptosis in Rats. Int. J. Mol. Sci..

[88] Abu-Elfotuh K., Abdel-Sattar S.A., Abbas A.N., Mahran Y.F., Alshanwani A.R., Hamdan A.M.E., Atwa A.M., Reda E., Ahmed Y.M., Zaghlool S.S. (2022). The Protective Effect of Thymoquinone or/and Thymol against Monosodium Glutamate-Induced Attention-Deficit/Hyperactivity Disorder (ADHD)-like Behavior in Rats: Modulation of Nrf2/HO-1, TLR4/NF-κB/NLRP3/Caspase-1 and Wnt/β-Catenin Signaling Pathways in Rat Model. Biomed. Pharmacother..

[89] Javed H., Azimullah S., Meeran M.F.N., Ansari S.A., Ojha S. (2019). Neuroprotective Effects of Thymol, a Dietary Monoterpene Against Dopaminergic Neurodegeneration in Rotenone-Induced Rat Model of Parkinson’s Disease. Int. J. Mol. Sci..

[90] Lee B., Yeom M., Shim I., Lee H., Hahm D.-H. (2020). Inhibitory Effect of Carvacrol on Lipopolysaccharide-Induced Memory Impairment in Rats. Korean J. Physiol. Pharmacol..

[91] Mahmoodi M., Amiri H., Ayoobi F., Rahmani M., Taghipour Z., Ghavamabadi R.T., Jafarzadeh A., Sankian M. (2019). Carvacrol Ameliorates Experimental Autoimmune Encephalomyelitis through Modulating Pro- and Anti-Inflammatory Cytokines. Life Sci..

[92] Borges R.S., Keita H., Ortiz B.L.S., Dos Santos Sampaio T.I., Ferreira I.M., Lima E.S., de Jesus Amazonas da Silva M., Fernandes C.P., de Faria Mota Oliveira A.E.M., da Conceição E.C. (2018). Anti-Inflammatory Activity of Nanoemulsions of Essential Oil from *Rosmarinus officinalis* L.: In Vitro and in Zebrafish Studies. Inflammopharmacology.

[93] Oualdi I., Brahmi F., Mokhtari O., Abdellaoui S., Tahani A., Oussaid A. (2021). Rosmarinus Officinalis from Morocco, Italy and France: Insight into Chemical Compositions and Biological Properties. Mater. Today Proc..

[94] Capatina L., Boiangiu R.S., Dumitru G., Napoli E.M., Ruberto G., Hritcu L., Todirascu-Ciornea E. (2020). Rosmarinus Officinalis Essential Oil Improves Scopolamine-Induced Neurobehavioral Changes via Restoration of Cholinergic Function and Brain Antioxidant Status in Zebrafish (*Danio rerio*). Antioxidants.

[95] Xu G., Guo J., Sun C. (2021). Eucalyptol Ameliorates Early Brain Injury after Subarachnoid Haemorrhage via Antioxidant and Anti-Inflammatory Effects in a Rat Model. Pharm. Biol..

[96] Bahrami T., Yaghmaei P., Yousofvand N. (2023). The Effects of Ibuprofen and 1, 8- Cineol on Anxiety and Spatial Memory in Hyperammonemic Rats. Metab. Brain Dis..

[97] Khan A., Vaibhav K., Javed H., Tabassum R., Ahmed M.E., Khan M.M., Khan M.B., Shrivastava P., Islam F., Siddiqui M.S. (2014). 1,8-Cineole (Eucalyptol) Mitigates Inflammation in Amyloid Beta Toxicated PC12 Cells: Relevance to Alzheimer’s Disease. Neurochem. Res..

[98] Habán M., Korczyk-Szabó J., Čerteková S., Ražná K. (2023). Lavandula Species, Their Bioactive Phytochemicals, and Their Biosynthetic Regulation. Int. J. Mol. Sci..

[99] López V., Nielsen B., Solas M., Ramírez M.J., Jäger A.K. (2017). Exploring Pharmacological Mechanisms of Lavender (*Lavandula angustifolia*) Essential Oil on Central Nervous System Targets. Front. Pharmacol..

[100] Caputo L., Piccialli I., Ciccone R., de Caprariis P., Massa A., De Feo V., Pannaccione A. (2021). Lavender and Coriander Essential Oils and Their Main Component Linalool Exert a Protective Effect against Amyloid-β Neurotoxicity. Phytother. Res..

[101] Hancianu M., Cioanca O., Mihasan M., Hritcu L. (2013). Neuroprotective Effects of Inhaled Lavender Oil on Scopolamine-Induced Dementia via Anti-Oxidative Activities in Rats. Phytomedicine.

[102] Sánchez-Vidaña D.I., Po K.K.-T., Fung T.K.-H., Chow J.K.-W., Lau W.K.-W., So P.-K., Lau B.W.-M., Tsang H.W.-H. (2019). Lavender Essential Oil Ameliorates Depression-like Behavior and Increases Neurogenesis and Dendritic Complexity in Rats. Neurosci. Lett..

[103] Ouanes S., Popp J. (2019). High Cortisol and the Risk of Dementia and Alzheimer’s Disease: A Review of the Literature. Front. Aging Neurosci..

[104] Li Y., Lv O., Zhou F., Li Q., Wu Z., Zheng Y. (2015). Linalool Inhibits LPS-Induced Inflammation in BV2 Microglia Cells by Activating Nrf2. Neurochem. Res..

[105] Sabogal-Guáqueta A.M., Osorio E., Cardona-Gómez G.P. (2016). Linalool Reverses Neuropathological and Behavioral Impairments in Old Triple Transgenic Alzheimer’s Mice. Neuropharmacology.

[106] Xu P., Wang K., Lu C., Dong L., Gao L., Yan M., Aibai S., Yang Y., Liu X. (2017). Protective Effects of Linalool against Amyloid Beta-Induced Cognitive Deficits and Damages in Mice. Life Sci..

[107] Galovičová L., Borotová P., Valková V., Vukovic N.L., Vukic M., Štefániková J., Ďúranová H., Kowalczewski P.Ł., Čmiková N., Kačániová M. (2021). *Thymus vulgaris* Essential Oil and Its Biological Activity. Plants.

[108] Horváth G., Horváth A., Reichert G., Böszörményi A., Sipos K., Pandur E. (2021). Three Chemotypes of Thyme (*Thymus vulgaris* L.) Essential Oil and Their Main Compounds Affect Differently the IL-6 and TNFα Cytokine Secretions of BV-2 Microglia by Modulating the NF-κB and C/EBPβ Signalling Pathways. BMC Complement. Med. Ther..

[109] Warman D.J., Jia H., Kato H. (2023). Effects of Thyme (*Thymus vulgaris* L.) Essential Oil on Aging-Induced Brain Inflammation and Blood Telomere Attrition in Chronologically Aged C57BL/6J Mice. Antioxidants.

[110] Capatina L., Todirascu-Ciornea E., Napoli E.M., Ruberto G., Hritcu L., Dumitru G. (2020). *Thymus vulgaris* Essential Oil Protects Zebrafish against Cognitive Dysfunction by Regulating Cholinergic and Antioxidants Systems. Antioxidants.

[111] Rekha K.R., Inmozhi Sivakamasundari R. (2018). Geraniol Protects Against the Protein and Oxidative Stress Induced by Rotenone in an In Vitro Model of Parkinson’s Disease. Neurochem. Res..

[112] Uddin M.S., Yu W.S., Lim L.W. (2021). Exploring ER Stress Response in Cellular Aging and Neuroinflammation in Alzheimer’s Disease. Ageing Res. Rev..

[113] El Azab E.F., Abdulmalek S. (2022). Amelioration of Age-Related Multiple Neuronal Impairments and Inflammation in High-Fat Diet-Fed Rats: The Prospective Multitargets of Geraniol. Oxid. Med. Cell. Longev..

[114] Liu Y., Zhou S., Huang X., Rehman H.M. (2022). Mechanistic Insight of the Potential of Geraniol against Alzheimer’s Disease. Eur. J. Med. Res..

[115] Hadian J., Hossein Mirjalili M., Reza Kanani M., Salehnia A., Ganjipoor P. (2011). Phytochemical and Morphological Characterization of Satureja Khuzistanica Jamzad Populations from Iran. Chem. Biodivers..

[116] Abbasloo E., Dehghan F., Khaksari M., Najafipour H., Vahidi R., Dabiri S., Sepehri G., Asadikaram G. (2016). The Anti-Inflammatory Properties of Satureja Khuzistanica Jamzad Essential Oil Attenuate the Effects of Traumatic Brain Injuries in Rats. Sci. Rep..

[117] Abbasloo E., Amiresmaili S., Shirazpour S., Khaksari M., Kobeissy F., Thomas T.C. (2023). Satureja Khuzistanica Jamzad Essential Oil and Pure Carvacrol Attenuate TBI-Induced Inflammation and Apoptosis via NF-κB and Caspase-3 Regulation in the Male Rat Brain. Sci. Rep..

[118] Abbasloo E., Khaksari M., Sanjari M., Kobeissy F., Thomas T.C. (2023). Carvacrol Decreases Blood-Brain Barrier Permeability Post-Diffuse Traumatic Brain Injury in Rats. Sci. Rep..

[119] Lee M., Lee S.H., Choi S., Choi B.Y., Suh S.W. (2022). Carvacrol Inhibits Expression of Transient Receptor Potential Melastatin 7 Channels and Alleviates Zinc Neurotoxicity Induced by Traumatic Brain Injury. Int. J. Mol. Sci..

[120] Ahmed A.B., Tahir H.M., Yousaf M.S., Munir F., Ali S. (2023). Efficacy of Silk Sericin and *Jasminum grandiflorum* L. Leaf Extract on Skin Injuries Induced by Burn in Mice. J. Burn Care Res..

[121] Sun L., Zhang Y., Wen S., Li Q., Chen R., Lai X., Zhang Z., Zhou Z., Xie Y., Zheng X. (2022). Extract of *Jasminum grandiflorum* L. Alleviates CCl4-Induced Liver Injury by Decreasing Inflammation, Oxidative Stress and Hepatic CYP2E1 Expression in Mice. Biomed. Pharmacother..

[122] Lu J., Zeng X., Feng Y., Li S., Wang Y., Liu Y., Chen F., Guan Z., Chen T., Wei F. (2023). Inhibitory Effects of *Jasminum grandiflorum* L. Essential Oil on Lipopolysaccharide-Induced Microglia Activation-Integrated Characteristic Analysis of Volatile Compounds, Network Pharmacology, and BV-2 Cell. Front. Pharmacol..

[123] Wang M., Tang H.-P., Wang S., Hu W.-J., Li J.-Y., Yu A.-Q., Bai Q.-X., Yang B.-Y., Kuang H.-X. (2023). Acorus Tatarinowii Schott: A Review of Its Botany, Traditional Uses, Phytochemistry, and Pharmacology. Molecules.

[124] Xu Z., Zhou X., Hong X., Wang S., Wei J., Huang J., Ji L., Yang Y., Efferth T., Hong C. (2023). Essential Oil of Acorus Tatarinowii Schott Inhibits Neuroinflammation by Suppressing NLRP3 Inflammasome Activation in 3 × Tg-AD Transgenic Mice. Phytomedicine.

[125] Ge Y., Zhen F., Liu Z., Feng Z., Wang G., Zhang C., Wang X., Sun Y., Zheng X., Bai Y. (2022). Alpha-Asaronol Alleviates Dysmyelination by Enhancing Glutamate Transport Through the Activation of PPARγ-GLT-1 Signaling in Hypoxia-Ischemia Neonatal Rats. Front. Pharmacol..

[126] Mikami M., Takuya O., Yoshino Y., Nakamura S., Ito K., Kojima H., Takahashi T., Iddamalgoda A., Inoue S., Shimazawa M. (2021). Acorus Calamus Extract and Its Component α-Asarone Attenuate Murine Hippocampal Neuronal Cell Death Induced by l-Glutamate and Tunicamycin. Biosci. Biotechnol. Biochem..

[127] Sen T., Saha P., Gupta R., Foley L.M., Jiang T., Abakumova O.S., Hitchens T.K., Sen N. (2020). Aberrant ER Stress Induced Neuronal-IFNβ Elicits White Matter Injury Due to Microglial Activation and T-Cell Infiltration after TBI. J. Neurosci..

[128] Jo M.-J., Kumar H., Joshi H.P., Choi H., Ko W.-K., Kim J.M., Hwang S.S.S., Park S.Y., Sohn S., Bello A.B. (2018). Oral Administration of α-Asarone Promotes Functional Recovery in Rats with Spinal Cord Injury. Front. Pharmacol..

[129] Yi M., Wang D., Chen Y., Xu X., Dai X. (2021). β-Asarone Suppresses TNF-α Expression through DNA Methylation and c-Jun-Mediated Transcription Modulation in Scratch-Injured Neuronal Cells. J. Biochem. Mol. Toxicol..

[130] Meng M., Zhang L., Ai D., Wu H., Peng W. (2021). β-Asarone Ameliorates β-Amyloid-Induced Neurotoxicity in PC12 Cells by Activating P13K/Akt/Nrf2 Signaling Pathway. Front. Pharmacol..

[131] Ning Z., Zhong X., Wu Y., Wang Y., Hu D., Wang K., Deng M. (2023). β-Asarone Improves Cognitive Impairment and Alleviates Autophagy in Mice with Vascular Dementia via the cAMP/PKA/CREB Pathway. Phytomedicine.

